# Supplementary material for: Fecal Luminal Factors from Patients with Gastrointestinal Diseases Alter Gene Expression Profiles in Caco-2 Cells and Colonoids
Source: Int J Mol Sci. 2022 Dec 7;23(24):15505. doi: 10.3390/ijms232415505 (PMC9779506; doi:10.3390/ijms232415505)
Supplement: Supplementary file 1 [file ijms-23-15505-s001.zip › ijms-2012865-supplementary.pdf]

Effects of luminal factors on the epithelial barrier in gastrointestinal diseases

Supplementary material

1 Supplementary methods

1.1 Liquid chromatography – mass spectrometry

Samples were prepared by extracting 30 µL of each sample with 270 µL of 90% MeOH (+4°C) in microcentrifuge tubes. The samples were then shaken vigorously and centrifuged for 10 min at 10 000 g (+4°C) after which the supernatants were transferred to chromatographic vials for analysis. Preparation of study-specific quality control samples (sQCs) was conducted by pooling equal amounts of samples in the first batch. The sQCs were subject to the same sample preparation procedure as the actual samples. sQCs were injected at the beginning, at the end, and systematically between every 11 samples throughout the batch sequence. The analysis of the samples was performed on an Agilent UHPLC-qTOF-MS system which consisted of a 1290 II Infinity series UHPLC system with a 6550 UHD iFunnel accurate-mass qTOF spectrometer. During the analysis, the samples were kept at 4°C. Analytes were separated by using both reversed phase (Waters Acquity UPLC HSS T3 column (100 × 2.1 mm, 1.8 µm)) and HILIC chromatography (Waters Acquity UPLC NH2, 100 x 2.1 mm, 1.7 µm). The Agilent MassHunter workstation was used to operate and monitor the instrument and acquire data. Reversed phase mobile phase included (A) water and (B) methanol, both containing 0.04% formic acid. The linear gradient elution was: 0 - 6 min, 5 – 100% B, 6 - 10.5 min, 100% B. The flow of mobile phase was set at 0.4 mL/min. HILIC mobile phases included (A) 10 mM ammonium formate in water and (B) 20 mM ammonium formate 90/10 (v/v) acetonitrile/water. The gradient was: 0 - 1 min 100 % B, 1 – 8 min 100 – 30 % B, 8 - 8.1 70 – 100 % B. Metabolites were ionized by a Jetstream electrospray ionization (ESI) source. The mass spectrometer was operated in both positive and negative modes and 1 and 2 µL of sample was injected for positive and negative modes, respectively. The spectrometer parameters were set as follows: drying gas (nitrogen) temperature at 175 °C and flow at 12 mL/min, sheath gas temperature at 350 °C and flow at 11 L/min, nebulizer pressure at 45 psi, capillary voltage at 3500 V, nozzle voltage at 300 V, fragmentor voltage at 175 V. Data were acquired within m/z 50 -1600 in centroid mode with the acquisition rate at 1.67 spectra/s. The MS abundance threshold was set at 200. Iterative MS/MS data acquisition was performed on sQC samples in both modes with 10, 20 and 40 eV collision energies and with the same chromatographic conditions as for the MS analysis.

The samples of each study group were analyzed in separate batches, which included its own quality control samples. The data-analytical workflow named "notame", described by Klåvus et al (1), was used to pre-process the acquired data and included drift correction within and between batches, data imputation was done using the *missForest* R package (2), and clustering of features was done to remove weak and repeated features (3).

1. Stekhoven DJ, Buhlmann P. Missforest-Non-Parametric Missing Value Imputation for Mixed-Type
Data. Bioinformatics (2012) 28(1):112-8. doi: 10.1093/bioinformatics/btr597.
2. Zheng R, Brunius C, Shi L, Zafar H, Paulson L, Landberg R, et al. Prediction and Evaluation of the Effect of Pre-Centrifugation Sample Management on the Measurable Untargeted Lc-Ms Plasma Metabolome. Anal Chim Acta (2021) 1182. doi: ARTN 33896810.1016/j.aca.2021.338968.

Log_{10} transformation was applied prior between-batch-correction to reduce possible batch effects caused by the instrument.

1.2 Establishment of a human colon-derived organoid culture

Sigmoid colonic biopsies (25-35 cm proximal from the anus) were obtained from a healthy volunteer, without prior bowel preparation, using standard biopsy forceps (Olympus, 3.3mm). Colonoid establishment protocol was modified from Co and colleagues(12) . Modification are described bellow:

The crypts were isolated from biopsies and embedded in Matrigel (Corning) containing 1 µM Jagged-1 peptide (AnaSpec, Fremont, CA, USA) and seeded in 24 well plates. The Matrigel simulates the extracellular matrix, to form 3D structures with the apical side facing inwards ("apical-in" colonoids). An organoid start medium kit was added to the wells, containing equal parts of IntestiCult™ OGM Human Component A and IntestiCult™ OGM Human Component B, Primocin (InvivoGen) diluted to 1:500 and p160ROCK inhibitor Y-27632 dihydrochloride (Tocris, Bristol, UK) diluted to 1:5000. The expansion medium, without Y-27632 dihydrochloride, was replaced every 2-3 days. When denser colonoid cultures had been stablished, colonoids were passaged every 7 days. All passages were performed by dissolving the Matrigel using Corning Cell Recovery Solution (Corning) and incubating the matrigel on ice. The dissolved matrigel was collected into Advanced DMEM/Ham's F-12 with 1x Glutamax (purchased from Gibco®), 10 mM HEPES (Gibco®) and 10% of heat-inactivated fetal bovine serum (FBS). The colonoid structures were disrupted by pipetting, and the fragments collected by centrifugation. Approximately 50 colonoids were used to seed each well. After passaging, cells were cultured in new matrigel and expansion medium as described above.

1.3 Immunofluorescence and imaging

Apical-in colonoids after dissolution of the Matrigel and apical-out colonoids after 72h in differentiation media were collected for staining. Content from each well was carefully transferred to conical tubes using 1000ml tips with enlarged ends. The tubes were centrifuged at 1100 rpm for 7 mins and after discarding the supernatant, the remaining material was carefully embedded in Tissue-Tek ®OCT™ compound (Sakura finetek, USA) and frozen in -80°C until sectioning on a microtome cryostat. Next, immunofluorescence staining was performed for phospho-ezrin in combination with Hoechst on the frozen cross-sections of the colonoids. Rabbit anti-phospho-Ezrin IgG (dilution 1:200) was used as the primary antibody (from Abcam, Cambridge, UK), while the secondary antibody was anti-rabbit Alexa 488 (dilution 1:200, Life Technologies). Lastly, Hoechst 33342 (dilution 1:10,000, ThermoFisher Scientific) was used to stain the nucleus of cells. Washes with PBS buffer followed every step described. Next, stained sections were mounted with ProLong Gold Antifade Mountant (ThermoFisher Scientific) on a glass slide. The preparations were kept sealed at 4°C until visualization. Images of the stained colonoid sections were acquired using 20x objective of a Carl Zeiss AxioImager.Z2 microscope (Carl Zeiss, Germany). Image analysis was performed using the Zeiss Zen 2 software (Carl, Zeiss). Images of the apical-in and apical-out colonoids during culture were taken in Leica DM IL LED Inverted Microscope using the 4x objective.

Table S1. Patient demographics and disease characteristics at inclusion

	Colon cancer	Ulcerative colitis	IBS	Healthy
Age - median (range)	80 (68-91)	52 (40-66)	37 (23-66)	32 (27-44)
Sex - male/female	3/3	3/3	3/3	3/3
Tumor staging* - I/II/III/IV	2/1/2/1	NA	NA	NA
Total Mayo score - median (range)	NA	9 (6-11)	NA	NA
Endoscopic Mayo score - mild/moderate/ severe inflammation	NA	0/4/2	NA	NA
IBS subtype (IBS-D/IBS-M/IBS-C)	NA	NA	4/2/0	NA
IBS-SSS - median (range)	NA	NA	278 (200-377)	NA

*According to TNM international classification

IBS, irritable bowel syndrome; IBS-D, irritable bowel syndrome with predominant diarrhea; IBS-M, irritable bowel syndrome with mixed bowel habits; IBS-C, irritable bowel syndrome with predominant constipation; IBS-SSS, IBS severity scoring system

Table S2. List of targeted genes included in the custom RT^2 PCR array **GENES RELATED TO ANTIMICROBIAL RESPONSE**

Gene name	Description
BIRC3	Baculoviral IAP repeat containing 3
CASP1	Caspase 1
CASP8	Caspase 8
CCL5[a]	Chemokine (C-C motif) ligand 5
CXCL1[a]	Chemokine (C-X3-C motif) ligand 1
CXCL2[a]	Chemokine (C-X3-C motif) ligand 2
DEFA5*#[a]	Defensin 5
DEFA6*[a]	Defensin 6
FADD	Fas (TNFRSF6)-associated via death domain
IL12A[a]	Interleukin 12A
IL12B*[a]	Interleukin 12B
IRF5	Interferon regulatory factor 5
IRF7	Interferon regulatory factor 7
JUN	Jun proto-oncogene
LYZ[a]	Lyzozyme
MYD88	Myeloid differentiation primary response gene (88)
RELA	V-rel reticuloendotheliosis viral oncogene homolog A
TLR1	Toll-like receptor 1
TLR2	Toll-like receptor 2
TLR3	Toll-like receptor 3
TLR4	Toll-like receptor 4
TLR5	Toll-like receptor 5
TLR6	Toll-like receptor 6
TLR9	Toll-like receptor 9

GENES RELATED TO METABOLITE SENSING

Gene name	Description
FFAR1*#	Free fatty acid receptor 1
FFAR2*#	Free fatty acid receptor 2
FFAR3*#	Free fatty acid receptor 3
O3FAR1	Omega-3 fatty acid receptor 1
FPR2*#	Formyl peptide receptor 2
HCAR2#	Hydroxycarboxylic acid receptor 2

GENES RELATED TO INFLAMMATORY RESPONSE

Gene name	Description
CCL2*	Chemokine (C-C motif) ligand 2
CCL20	Chemokine (C-C motif) ligand 20
CCL25#	Chemokine (C-C motif) ligand 25
CCL5[a]	Chemokine (C-C motif) ligand 5
CX3CL1	Chemokine (C-X3-C motif) ligand 1
CXCL1[a]	Chemokine (C-X-C motif) ligand 1
CXCL10	Chemokine (C-X-C motif) ligand 10
CXCL11*	Chemokine (C-X-C motif) ligand 11
CXCL2[a]	Chemokine (C-X-C motif) ligand 2
CXCL9	Chemokine (C-X-C motif) ligand 9
CXCR3	Chemokine (C-X-C motif) receptor 3
DEFA5*#	Defensin 5
DEFA6*	Defensin 6
IFNG*#	Interferon gamma
IL12A[a]	Interleukin 12A
IL17A*#	Interleukin 17A
IL1B	Interleukin 1 beta
IL1RL1*#	Interleukin 1 receptor-like 1
IL23A[a]	Interleukin 23, alpha subunit p19
IL33	Interleukin 33
IL6#[a]	Interleukin 6
LYZ	Lyzozyme
NFKB1	Nuclear factor of kappa B subunit 1
NFKBIA	Nuclear factor of kappa B inhibitor alpha
NOS2[a]	Nitric oxide synthase 2
PECAM1	Platelet/endothelial cell adhesion molecule
PTGS2[a]	Prostaglandin-endoperoxide synthase 2
S100A8*#	S100 calcium binding protein A8
S100A9	S100 calcium binding protein A9
STAT1[a]	Signal transducer and activator of transcription 1
STAT3[a]	Signal transducer and activator of transcription 3
TNF[a]	Tumor necrosis factor
IL8	Interleukin 8

GENES RELATED TO CANCER IMMUNITY AND CARCINOGENESIS

Gene name	Description
BCL2	B-cell lymphoma 2
CCL2*	Chemokine (C-C motif) ligand 2
CCL20	Chemokine (C-C motif) ligand 20
CXCL1	Chemokine (C-X-C motif) ligand 1
CXCL10	Chemokine (C-X-C motif) ligand 10
CXCL9	Chemokine (C-X-C motif) ligand 9
IFNG*#	Interferon gamma
IL12A[a]	Interleukin 12A
IL12B*#[a]	Interleukin 12B
IL23A[a]	Interleukin 23, alpha subunit p19
IL6#[a]	Interleukin 6
MYD88[a]	Myeloid differentiation primary response gene (88)
NOS2[a]	Nitric oxide synthase 2
PTGS2[a]	Prostaglandin-endoperoxide synthase 2
STAT1[a]	Signal transducer and activator of transcription 1
STAT3[a]	Signal transducer and activator of transcription 3
TNF[a]	Tumor necrosis factor
TP53	Tumor protein p53
TRAF6	TNF receptor-associated factor 6

GENES RELATED TO EPITHELIAL BARRIER INTEGRITY

Gene name	Description
CLDN1	Claudin 1
CLDN15	Claudin 15
CLDN2	Claudin 2
CLDN6	Claudin 6
DSC2	Desmocolin 2
EGFR	Epidermal growth factor receptor
ICAM1	Intercellular adhesion molecule 1
MUC1	Mucin 1
NOTCH1	Notch 1
NOTCH2	Notch 2
OCLN	Occludin

ADDITIONAL GENES

Gene name	Description
ALOX12	Arachidonate 12-lipoxygenase
ALOX15	Arachidonate 15-lipoxygenase
ALOX15B	Arachidonate 15-lipoxygenase type B
ANXA1	Annexin A1
CD1D	CD1d molecule
IFNA1	Interferon, alpha 1
IFNB1*#	Interferon, beta 1
IL10*#	Interleukin 10
IL10RA1	Interleukin 10 receptor alpha
IL13	Interleukin 13
CHGA#	Chromogranin A
TGFB1	Transforming growth factor beta 1
TLR7#	Toll-like receptor 7
TLR8*#	Toll-like receptor 8
TNFSF13	Tumor necrosis factor (ligand) superfamily member 13
TOLLIP	Toll interacting protein
TSLP#	Thymic stromal lymphopoietin

HOUSEKEEPING GENES

Gene name	Description
GAPDH	Glyceraldehyde-3-phosphate dehydrogenase
B2M	Beta-2-microglobulin
RPLP0	Ribosomal protein, large P0
HPRT1	Hypoxanthine phosphoribosyltransferase 1
ACTB	Actin beta

*Gene excluded from Caco-2 cell analysis

#Gene excluded from the colonoid analysis

[a] Genes shared by more than one category

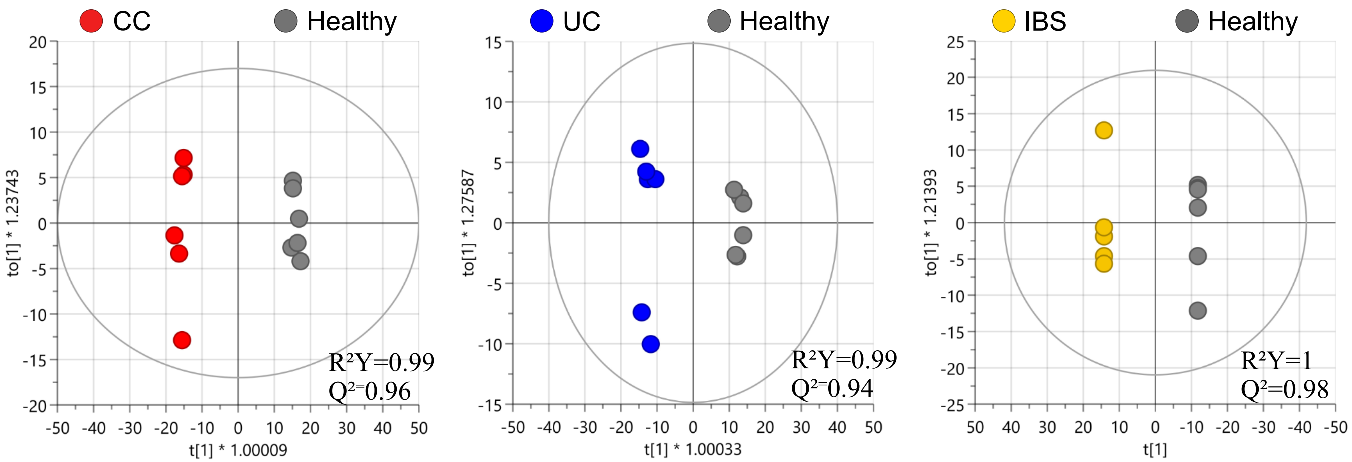

Figure S1. Metabolite profiles of fecal supernatants from healthy subjects and patients with colon cancer (CC), ulcerative colitis (UC) and irritable bowel syndrome (IBS). Fecal supernatants were analyzed by untargeted LC/MS. Results for the detected 9,699 spectral features of the fecal supernatants were analyzed by orthogonal partial least squares - discriminant analysis (OPLS-DA) comparing each of the patient groups with healthy subjects. Score scatter plots from OPLS-DA using a VIP cut-off>1.5 comparing (A) CC patients with healthy subjects, (B) UC patients with healthy subjects, and (C) IBS patients with healthy subjects . N=6 in each group. CC, colon cancer; UC, ulcerative colitis; IBS, irritable bowel syndrome.

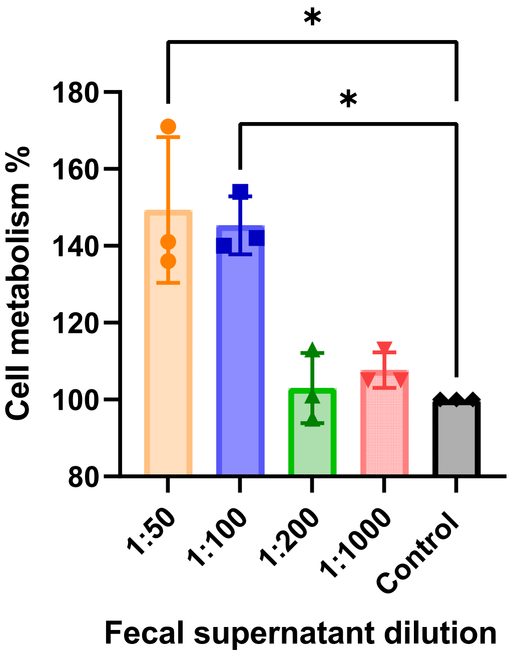

Figure S2. Caco-2 cell metabolism in co-culture with fecal supernatants. Caco-2 cells were seeded in 96-well, flat bottom plates and cultured for 48h with fecal supernatants diluted in cell culture media at 1:50, 1:100, 1:200 and 1:1000 concentrations. Cell metabolism was measured by a colorimetric assay based on the MTT labeling reagent. Values are expressed as percentages of metabolically active cells found in stimulated cells in relation to the average of untreated controls. Statistical differences between the groups were assessed by the Kruskal-Wallis test followed by Dunn’s multiple comparisons test. (N=3 for each group).

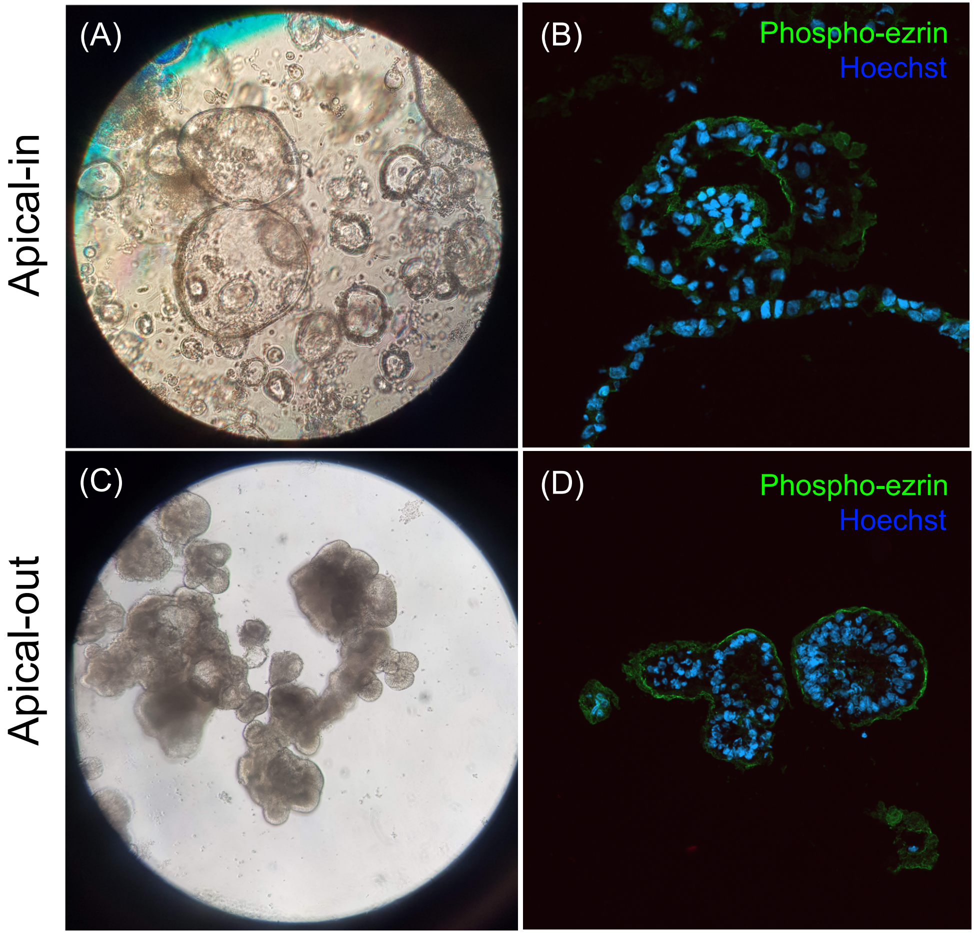

Figure S3. Characterization of apical-out colonoids. Colonoids obtained from colonic biopsies from a healthy donor were grown embedded in Matrigel for culture expansion. The Matrigel is later dissolved and the colonoids are cultured in suspension in differentiation media for 72h. Images show apical-in colonoids (top row) and apical-out colonoids (bottom row). Light microscopy at 4x magnification of (A) apical-in colonoids immediately after suspension in differentiation media, and (C) apical-out colonoids 72h later in differentiation media. Corresponding immunofluorescence cross-section images showing polarized apical surface of the cells defined by phosphor-ezrin (green) in (B) apical-in colonoids before differentiation and(D) differentiated apical-out colonoids; 20x magnification.
